# 

**Published:** 1877-08

**Authors:** 


### CONTENTS:

ARTICLE I.—The Principles Involved in the Treatment of Diseased Conditions. By F. Peabody, M. D. .... 145

ARTICLE II.—Dr. Chase and his Theories. By J. Campbell .... 157

ARTICLE III—Principles Involved in Filling Teeth with Gold Foil. By S. B. Palmer, D. D. S .... 164

ARTICLE IV.—Meetings of American Dental Convention, Southern Dental Association, etc .... 167

ARTICLE V.—The Etiology of Alcoholic Degeneration .... 170

ARTICLE VI.—Metallotherapy .... 173

ARTICLE VII.—Periosteal Surgery in the United States .... 176

ARTICLE VIII.—Death while under Ether .... 178

ARTICLE IX.—Dental Alloys. By Henry S. Chase, M. D., D. D. S .... 179

ARTICLE X.—North Carolina State Dental Association .... 181

### EDITORIAL, ETC.

The Johns Hopkins Hospital of Baltimore City .... 182
Homœopathy in Danger .... 184

### MONTHLY SUMMARY.

Excision of the Inferior Maxilla .... 185
Excision of the Branches of the Fifth Pair for Neuralgia .... 186
The Length of the Fraenum of the Tongue .... 187
The Treatment of Atheromatous Cysts of the Neck .... 187
Injection of Carbolic Acid for Neuralgia .... 188
Anæsthesia .... 188
Local Anæsthesia by Bromhydrate of Quinine .... 189
Bromide of Potassium as a Caustic .... 189
Death during Etherization .... 190
Phenicated Camphor .... 190
Poisonous Properties of Glycerine .... 190
Death from Chloroform .... 191
Suppression of the Salivary Secretion .... 191
Nussbaum's Narcosis .... 191
Russian Female Medical School .... 192
Properties of the Human Gastric Juice .... 192

